# Invasion of shoot apical meristems by *Chrysanthemum stunt viroid* differs among *Argyranthemum* cultivars

**DOI:** 10.3389/fpls.2015.00053

**Published:** 2014-02-16

**Authors:** Zhibo Zhang, YeonKyeong Lee, Carl Spetz, Jihong Liu Clarke, Qiaochun Wang, Dag-Ragnar Blystad

**Affiliations:** ^1^State Key Laboratory of Crop Stress Biology for Arid Areas, Key Laboratory of Genetic Improvement of Horticultural Crops of Northwest China, Ministry of Agriculture of China – College of Horticulture, Northwest A&F University, YanglingChina; ^2^Bioforsk-Norwegian Institute for Agricultural and Environmental Research, ÅsNorway; ^3^Department of Plant Sciences, Norwegian University of Life Sciences, ÅsNorway

**Keywords:** *Argyranthemum*, callose, *Chrysanthemum stunt viroid* (CSVd), *in situ* hybridization, plasmodesmata, shoot apical meristem

## Abstract

*Chrysanthemum stunt viroid* (CSVd) is a damaging pathogen attacking *Argyranthemum* plants. Our study attempted to reveal distribution patterns of CSVd in shoot apical meristems (SAM) and to explore reasons for differential ability of CSVd to invade SAM of selected *Argyranthemum* cultivars. Symptom development was also observed on greenhouse-grown *Argyranthemum* plants. Viroid localization using *in situ* hybridization revealed that the ability of CSVd to invade SAM differed among cultivars. In diseased ‘Yellow Empire’ and ‘Butterfly’, CSVd was found in all tissues including the uppermost cell layers in the apical dome (AD) and the youngest leaf primordia 1 and 2. In diseased ‘Border Dark Red’ and ‘Border Pink’, CSVd was detected in the lower part of the AD and elder leaf primordia, leaving the upper part of the AD, and leaf primordia 1 and 2 free of viroid. Histological observations and transmission electron microscopy showed similar developmental patterns of vascular tissues and plasmodesmata (PD) in the SAM of ‘Yellow Empire’ and ‘Border Dark Red’, while immunolocalization studies revealed a major difference in the number of callose (β-1, 3-glucan) particles deposited at PD in SAM. A lower number of callose particles were found deposited at PD of SAM of ‘Yellow Empire’ than ‘Border Dark Red’. This difference is most likely responsible for the differences in ability of CSVd to invade SAM among *Argyranthemum* cultivars.

## INTRODUCTION

Viroids consist of small (246–401 nucleotides), single-stranded and circular RNA molecules (Flores et al., 2005), and cause severe damage to plants. CSVd, a member of the genus *Pospiviroid*, family *Pospiviroidae* (Lawson, 1987; King et al., 2012), can attack several flower plant species such as *Chrysanthemum* (Diener and Lawson, 1973; Horst et al., 1977; Bouwen and Van Zaayen, 2003)*, Argyranthemum* (Menzel and Maiss, 2000; Marais et al., 2011; Torchetti et al., 2012), *Dahlia* (Nakashima et al., 2007), and *Petunia* (Verhoeven et al., 1998). CSVd has been included in the EPPO A2 list of quarantine pathogens (OEPP/EPPO, 2014). CSVd infection causes various adverse effects on diseased *Chrysanthemum* plants including stunted growth, short internodes, poor root development, reduced flower size, and flower color bleaching, consequently resulting in the production of unmarketable plants and low yield of flowers (Horst et al., 1977; Chung et al., 2001; Jeon et al., 2012; Matsushita, 2013; Savitri et al., 2013). Symptoms such as yellow deformed leaves with terminal necrosis, flower distortion, or leaf necrosis were observed on CSVd-infected *Argyranthemum* ‘Butterfly’ plants (Marais et al., 2011). Interestingly, CSVd has recently been found to alter the photoperiodic response of the diseased *Chrysanthemum* plants. Under long-day conditions, CSVd-infected *Chrysanthemum* plants flowered autonomously whereas CSVd-free plants maintained their normal vegetative growth (Hosokawa et al., 2004a).

For vegetatively propagated plants including *Chrysanthemum* and *Argyranthemum*, viroids are transmitted from generation to generation, resulting in production of contaminated plant materials (Chung et al., 2009). Use of viroid-free plants is pivotal for a sustainable production of these plants and the exchange of materials between countries. To date, various methods have been developed for production of viroid-free plants, including a combination of meristem culture with either high (Hollings and Stone, 1970; Stace-Smith and Mellor, 1970; Jeon et al., 2012) or low temperature therapy (Lizárraga et al., 1980; Paludan, 1985; Paduch-Cichal and Kryczyński, 1987; Savitri et al., 2013), chemotherapy (Horst and Cohen, 1980; Savitri et al., 2013), and LP-free SAM culture (Hosokawa et al., 2004b,c). CSVd-free plants can be obtained by meristem-culture based methods. However, it is common that the size of the shoot tip required to obtain CSVd-free plants differs between plant cultivars (Hosokawa et al., 2004c; Chung et al., 2006; Jeon et al., 2012; Kovalskaya and Hammond, 2014). These data suggest that invasion of the meristematic tissue by CSVd might differ between plant cultivars. However, experimental evidence for this is still lacking, and explanation for this has remained unclear.

The present study first identified distribution patterns of CSVd in SAM of four *Argyranthemum* cultivars, and found that the ability of CSVd to invade SAM differed among the cultivars. Therefore, we further explored causes responsible for this difference. Symptom development on greenhouse-grown CSVd-infected plants was also observed.

## MATERIALS AND METHODS

### PLANT MATERIALS

*Argyranthemum* ‘Yellow Empire’, ‘Border Dark Red’, ‘Butterfly’ and ‘Border Pink’, which were infected with CSVd, were included in the present study. ‘Yellow Empire’ and ‘Border Dark Red’ were used in all the experiments, including *in situ* localization of CSVd, histological observations on vascular bundles in SAM and immunolocalization of callose, while ‘Butterfly’ and ‘Border Pink’ were only used in *in situ* localization of CSVd. No healthy plants of ‘Yellow Empire’, ‘Butterfly’, or ‘Border Pink’ were available and only healthy ‘Border Dark Red’ plants were included. All stock plants were screened for CSVd using RT-PCR in order to confirm CSVd infection (see below). All CSVd-infected stock plants were maintained in net-screened greenhouse conditions.

### DETECTION OF CSVd BY RT-PCR

Total RNA was isolated from 100 mg of leaf tissue using the Plant RNA Mini Kit (Omega Bio-Tek, USA) following the manufacturer’s instruction. RT was performed with Superscript II Enzyme (Invitrogen, USA) using 1 μg of total RNA, according to Zhang et al. (2014). After RT reaction, PCR amplification was performed in a C1000TM thermal cycler (BIO-RAD, Singapore), using Tfi polymerase (Invitrogen, USA) and CSVd specific primers (forward primer 5^′^–3^′^ CGGGACTTACTGTGGTTCC and reverse primer 5^′^–3^′^ GGAAGGGTGAAAACCCTGTT; Zhang et al., 2014). PCR was conducted by subjecting the samples to the following conditions: initial denaturation for 2 min at 95°C, followed by 35 cycles of 95°C for 20 s, 58°C for 30 s and 70°C for 30 s, and a final extension for 7 min at 70°C. PCR products were separated on a 1% agarose gel and visualized under UV light.

### OBSERVATIONS OF SYMPTOM DEVELOPMENT

Cuttings taken from CSVd-infected ‘Yellow Empire’ and ‘Border Dark Red’, and healthy ‘Border Dark Red’ stock plants were rooted and grown for vegetative and flower production in greenhouse conditions, according to Zhang et al. (submitted). Symptom development was observed during the whole procedure of plant production.

### PROBE SYNTHESIS

A recombinant plasmid (PCSVd2) containing a portion of the CSVd genome was used for *in vitro* transcription. This construct was generated by amplifying a 188 nucleotides fragment of the CSVd genome using the forward primer (5^′^–3^′^ CGGGACTTACTGTGGTTCC) and the reverse primer (5^′^–3^′^ GGAAGGGTGAAAACCCTGTT) and cloning it into the pGEM®;-T easy vector (Promega, USA). Sequencing of the plasmid with M13 primers was performed in order to confirm the orientation of the CSVd insert (GATC Biotech). Briefly, digoxigenin (DIG)-11-UTP probes were generated by linearizing the plasmid with the appropriate restriction enzyme followed by a transcription reaction with T7 or SP6 RNA polymerase to generate sense and antisense probes, respectively, following the manufacturer’s instructions (Roche, Germany). After the transcription and DNAse treatment (Promega, USA), the RNA was ethanol precipitated overnight at -20°C and re-suspended in RNase free water. Incorporation and quantification of the DIG label into the transcripts was verified by dot blot analysis with alkaline phosphatase-conjugated anti-DIG antibody (Roche, Germany) as specified by the manufacturer.

### *IN SITU* HYBRIDIZATION

Shoot apical meristem (about 3 mm in size) containing 6–7 LPs were excised from healthy ‘Border Dark Red’, and CSVd-infected ‘Border Dark Red’, ‘Yellow Empire’, ‘Butterfly’, and ‘Border Pink’ stock plants. The samples were fixed, dehydrated and paraplast embedded, according to Lee et al. (2008). Sections (10 μm thick) were cut with a Rotary Microtome (Leica RM 2255, Germany), collected onto a poly-l-lysine coated slide glass (Thermo Scientific, Germany). *In situ* hybridization was performed as described by Rodio et al. (2007). Briefly, DIG-labeled RNA probes were added to the hybridization solution containing 50% formamide, 10% dextran sulfate, 5x Denhardt’s solution (1x Denhardt’s solution contains 0.02% Ficoll, 0.02% polyvinylpyrrolidone, and 0.02% bovine serum albumin), 1 mg ml^-1^ tRNA (Sigma, USA), 300 mM NaCl, 10 mM Tris-HCl, pH 6.8, 10 mM saline phosphate buffer, and 5 mM EDTA. The hybridization (carried out overnight) and the three subsequent washes (for 30, 90, 60 min in a buffer consisting of 2x SSC buffer and 50% formamide) were all performed at 70°C. Then the sections were placed in blocking solution (Roche, Germany) for 1 h, followed by incubation for 2 h at room temperature with alkaline phosphatase-conjugated anti-DIG antibody (1:2000 dilution) in blocking solution. The sections were then washed in TBS buffer (100 mM Tris-HCl, pH 7.5, 400 mM NaCl and 0.1% Tween 20) for three times at room temperature, 20 min each, and incubated in alkaline phosphate buffer for 5 min. Color reaction was performed using the substrate solution (nitro-blue tetrazolium chloride/5-bromo-4-chloro-3-indolyphosphate *p*-toluidine salt, NBT/BCIP; Promega, USA) in the dark. Results were examined with a light microscope (Leica, Germany).

### HISTOLOGICAL OBSERVATIONS AND TRANSMISSION ELECTRON MICROSCOPY

Shoot apical meristem (about 3 mm) containing 6–7 LPs were excised from healthy ‘Border Dark Red’, CSVd-infected ‘Border Dark Red’ and ‘Yellow Empire’, and fixed as described in Lee et al. (2008). After dehydration, the samples were embedded in LR White resin (London Resin Company, England). For histological observations of vascular development, thick sections (1 μm) were cut with an ultra-microtome (EM UC6, Leica, Germany). The sections were stained with Stevenel’s Blue and observed under a light microscope (Leica, Germany). Ultra-thin sections (70–80 nm) were also obtained using the ultra-microtome. Some of the ultra-thin sections were mounted on formvar coated copper slot grids (Electron Microscopy Sciences, USA) and stained with a mixture of 4% uranyl acetate (Polysciences, Inc, USA) and 1% potassium permanganate for 8 min, and examined under a transmission electron microscope (Morgagni 268, FEI Company B.V., The Netherlands) for observations of PD. Other similar ultra-thin sections were mounted on formvar and carbon-coated nickel grids (100 mesh; Electron Microscopy Sciences, USA) and were used for immunolocalization of callose (β-1, 3-glucan; see below).

### IMMUNOLOCALIZATION OF CALLOSE

Ultra-thin sections (70–80 nm) that were mounted on formvar and carbon-coated nickel grids were used for immunolocalization of callose at PD, according to Lee et al. (2008), with some modifications. In brief, the sections were blocked with 3% bovine serum albumin in phosphate-buffered saline (BSA/PBS) for 30 min. The sections were incubated in a primary monoclonal antibody (1:500 dilution in BSA/PBS) against β-1, 3-glucans (Bio-supplies, Parkville, VIC, Australia) for 1 h, followed by three washes with PBS, 5 min each. Anti-mouse IgG (whole molecule)-gold (10 nm; Sigma, USA) was applied as a secondary antibody for 1 h, followed by three washes with PBS, 5 min each. Negative control was done without the primary antibody. Immunolabeled sections were stained using a mixture of 4% uranyl acetate and 1% potassium permanganate for 8 min and observed under a transmission electron microscope (Morgagni 268, FEI Company B.V., The Netherlands).

## RESULTS

### SANITARY STATUS OF THE STOCK PLANTS

**FIGURE 1:**
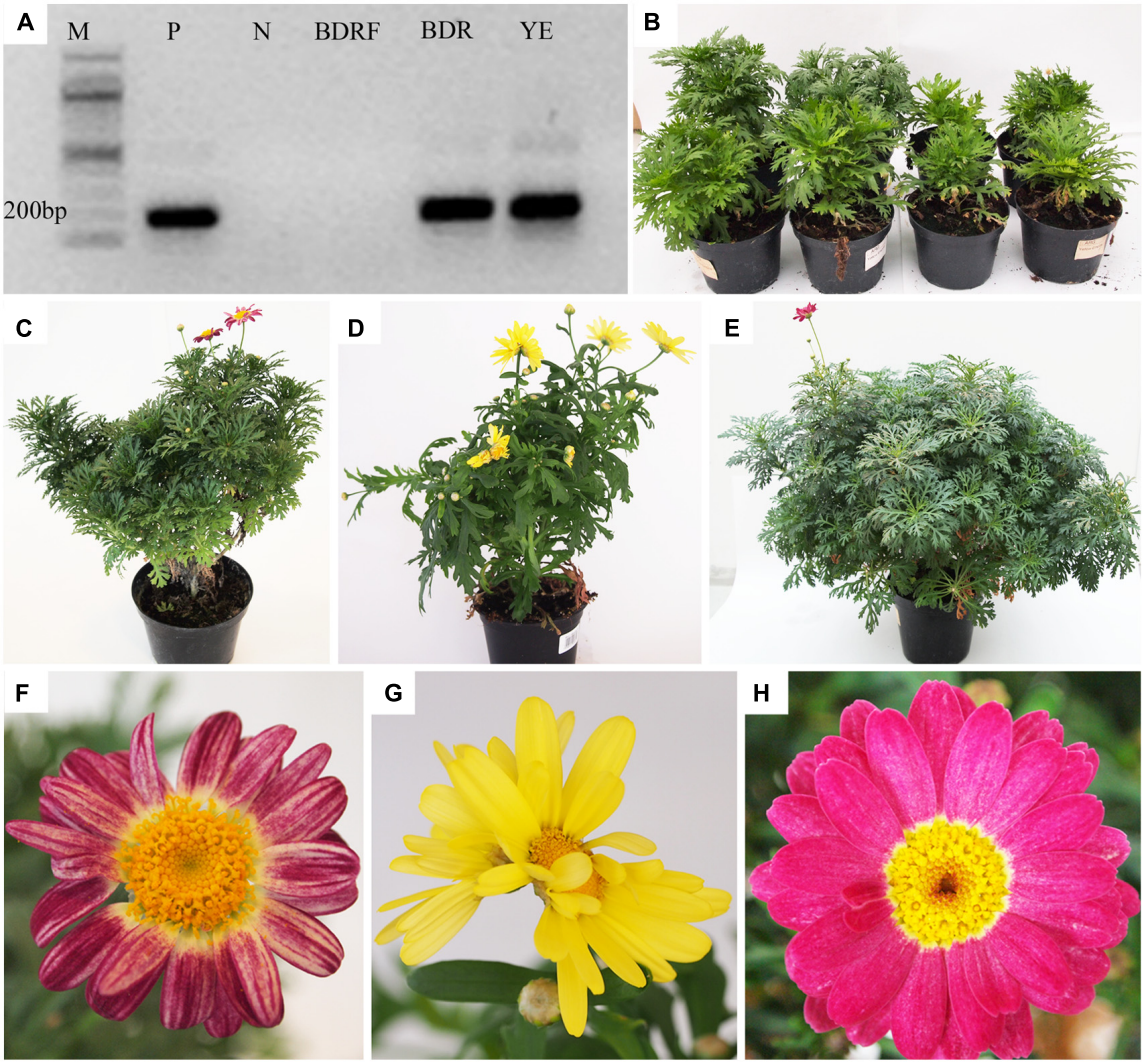
**Detection of *Chrysanthemum stunt viroid* (CSVd) and symptoms in CSVd-infected *Argyranthemum* plants. (A)** CSVd detection using RT-PCR. **(B)** Variation in growth of ‘Border Dark Red’. The left two plants are healthy and right two are CSVd-infected plants. **(C)** CSVd-infected ‘Border Dark Red’ plant. **(D)** CSVd-infected ‘Yellow Empire’ plant. **(E)** Healthy ‘Border Dark Red’ plant. **(F)** Abnormal flower of CSVd-infected ‘Border Dark Red’ plant. **(G)** Abnormal flower of CSVd-infected ‘Yellow Empire’. **(H)** Normal flower of healthy ‘Border Dark Red’ plant. *M* = 100 bp DNA ladder; P = positive control of CSVd; *N* = Milli-Q water; BDRF = RNA from healthy ‘Border Dark Red’; BDR = RNA from CSVd-infected ‘Border Dark Red’; YE = RNA from CSVd-infected ‘Yellow Empire’.

Systemic infection of the stock plants used in the present study was verified by RT-PCR (**Figure 1A**). CSVd-specific fragments of about 200 bp were detected in all the diseased stock plants of ‘Yellow Empire’, ‘Border Dark Red’, ‘Butterfly’ and ‘Border Pink’, indicating that these plants were CSVd-infected. No such bands were found in healthy plants of ‘Border Dark Red’ (**Figure 1A**).

### SYMPTOM DEVELOPMENT

Reduced vegetative growth was found in CSVd-infected ‘Border Dark Red’ plants, resulting in production of one-half or two-third size of the healthy plants (**Figure 1B**). The diseased plants of ‘Border Dark Red’ (**Figure 1C**) and ‘Yellow Empire’ (**Figure 1D**) displayed irregular shape, compared with the healthy plants of ‘Border Dark Red’ (**Figure 1E**). Flower distortion and color breaking were observed in the infected ‘Border Dark Red’ (**Figure 1F**) and ‘Yellow Empire’ (**Figure 1G**), while the healthy ‘Border Dark Red’ plants produced normal flowers (**Figure 1H**).

### DISTRIBUTION OF CSVd IN SAM DIFFERS AMONG CULTIVARS

**FIGURE 2:**
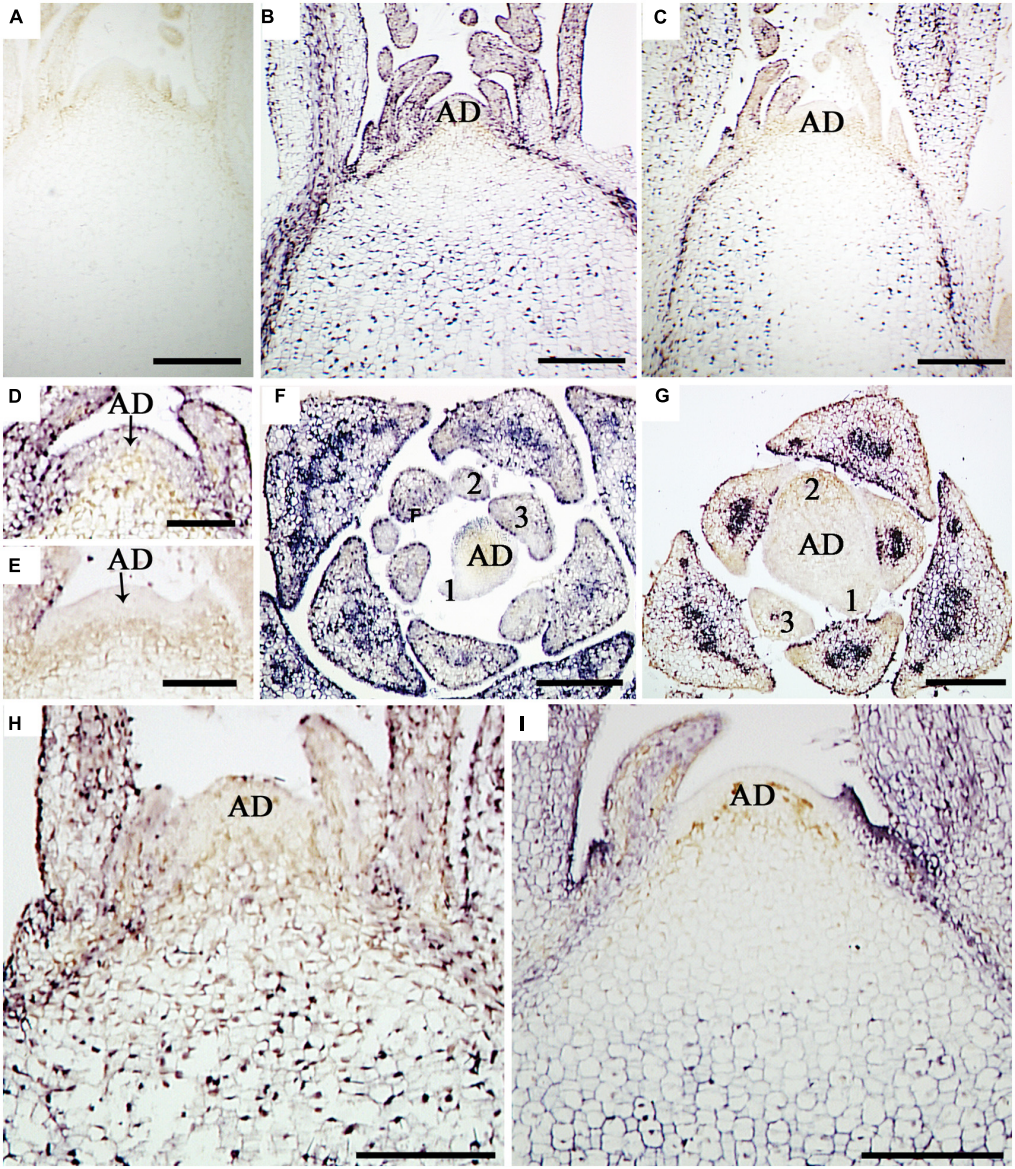
***In situ* localization of CSVd in SAM of the CSVd infected *Argyranthemum* plants. (A)** Longitudinal sections of healthy shoot tips of ‘Border Dark Red’. **(B)** Longitudinal section of CSVd-infected shoot tip of ‘Yellow Empire’. **(C)** Longitudinal section of CSVd-infected shoot tip of ‘Border Dark Red’. **(D)** Longitudinal section of CSVd-infected SAM of ‘Yellow Empire’ [higher magnification of the SAM in **(B)**]. **(E)** Longitudinal section of CSVd-infected shoot apical meristem (SAM) of ‘Border Dark Red’ [higher magnification of the SAM in **(C)**]. **(F)** Cross section of CSVd-infected shoot tip of ‘Yellow Empire’. **(G)** Cross section of CSVd-infected shoot tip of ‘Border Dark Red’. **(H)** Longitudinal section of CSVd-infected shoot tip of ‘Butterfly’. **(I)** Longitudinal section of CSVd-infected shoot tip of ‘Border Pink’. AD indicates apical dome (AD), and 1, 2, 3 indicates first, second, and the third leaf primordia, respectively. Scale bars in **(A**, **B**, **C**, **H**, **I)** are 310 μm; scale bars in **(D–G)** are 100 μm.

*In situ* hybridization with strand-specific DIG-labeled CSVd antisense-probes resulted in purple-blue color reaction (viroid) in the SAM cells of CSVd-infected samples (**Figures 2B,C,H,I**), while no such color reactions were seen in the healthy sample (**Figure 2A**), indicating efficient detection of the viroid. *In situ* hybridization of CSVd-infected ‘Yellow Empire’, strong viroid signals were revealed throughout SAM (**Figure 2B**), including AD, and the youngest LP 1 and 2 (**Figures 2D,F**). Similar localization patterns were observed in ‘Butterfly’ (**Figure 2H**). In the case of diseased ‘Border Dark Red’, CSVd was easily detected in the lower parts of AD, in LP 3 and elder tissues of SAM (**Figure 2C**). However, no viroid was detected in the uppermost section of AD, and LP 1 and 2 (**Figures 2E,G**).. The viroid-free area of AD in ‘Border Dark Red’ contained about 20 layers of cells, being approximately 0.2 mm in size. Similar patterns of CSVd distribution were observed in ‘Border Pink’ (**Figure 2I**). In this experiment, at least 15 SAM of each cultivar were observed.

### SIMILAR DEVELOPMENT PATTERN OF VASCULAR BUNDLES IN SAM OF ‘YELLOW EMPIRE’ AND ‘BORDER DARK RED’

**FIGURE 3:**
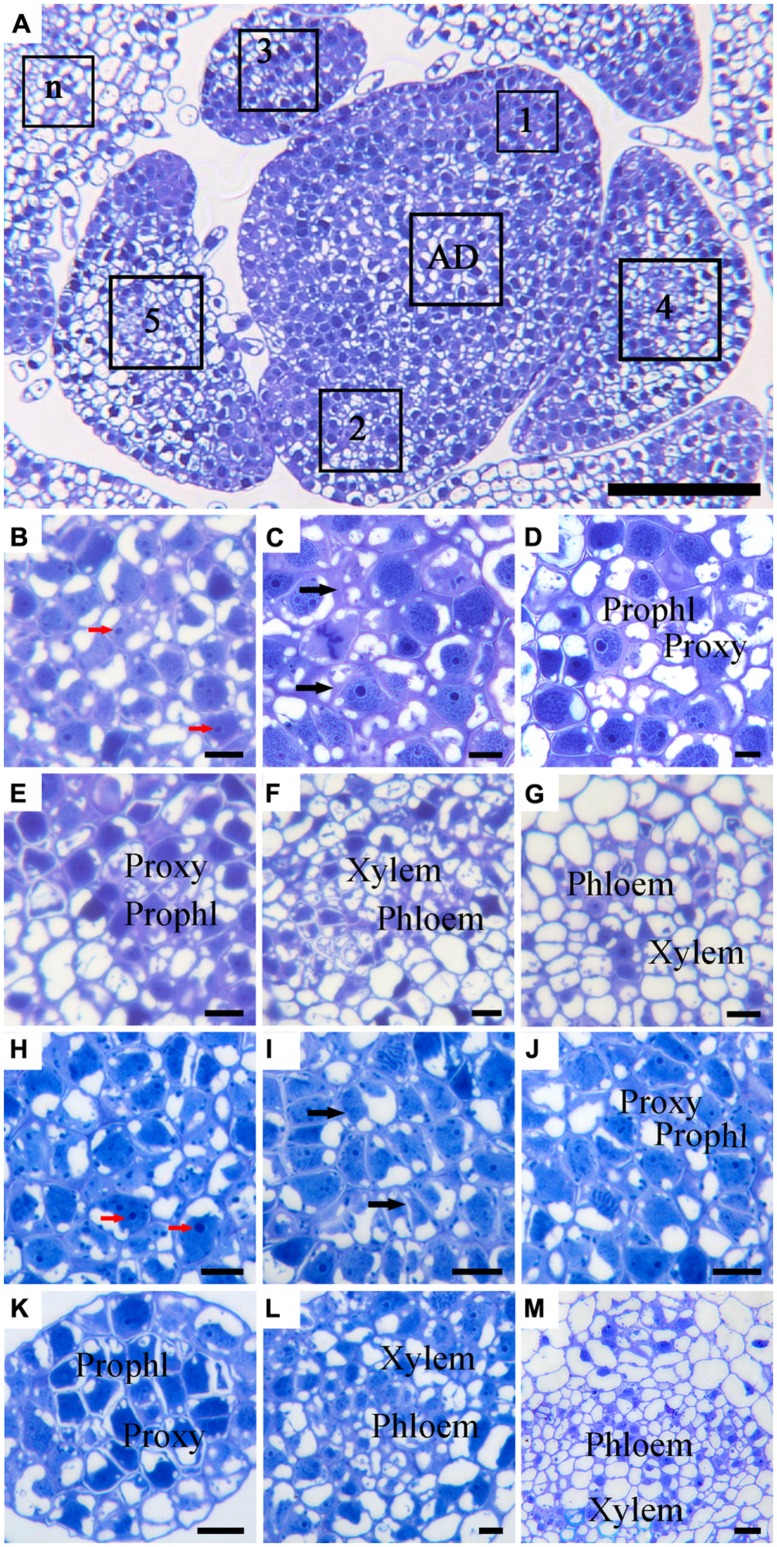
**Histological observation of cell structures and phloem in cross-sectioned SAM of ‘Border Dark Red’ and ‘Yellow Empire’. (A)** An overview of the cross-section of the AD in the meristem and the young leaf primordia (LP; 1, 2, 3, 4, 5 and n) of ‘Border Dark Red’. **(B–G)** High magnification of the LPs in **(A)** (AD and LP1, 2, 3, 4, 5, respectively). **(H–M)** High magnification of the LPs of ‘Yellow Empire’ shoot tip cross-section (AD and LP1, 2, 3, 4, 5, respectively). Prophl, prophloem; Proxy, proxylem. Red arrows indicate densely stained nucleolus. Black arrows indicate cell walls. Scale bar in **(A)** is 100 μm; **(B–M)** are 10 μm.

Due to the alternate phyllotaxy pattern in *Argyranthemum* plants, cross sections were cut to allow observations on the AD and all LPs simultaneously (**Figure 3A**). In both ‘Yellow Empire’ and ‘Border Dark Red’, cells in the AD had large nuclei, densely stained nucleolus and thin cell wall, and were isodiametric in shape (**Figures 3B,H**). In LP1, cells were less regular in size and had thick cell walls, but vascular tissues had not yet developed (**Figures 3C,I**). However, in LP2 (**Figures 3D,E**) and LP3 (**Figures 3J,K**), cells were significantly differentiated, and both proxylem and prophloem tissues were already developed. In LP4 and elder tissues, complex vascular bundles including xylem and phloem were clearly seen (**Figures 3F,G,L,M**).

### SIMILAR ULTRASTRUCTURE OF PD IN SAM OF ‘YELLOW EMPIRE’ AND ‘BORDER DARK RED’

**FIGURE 4:**
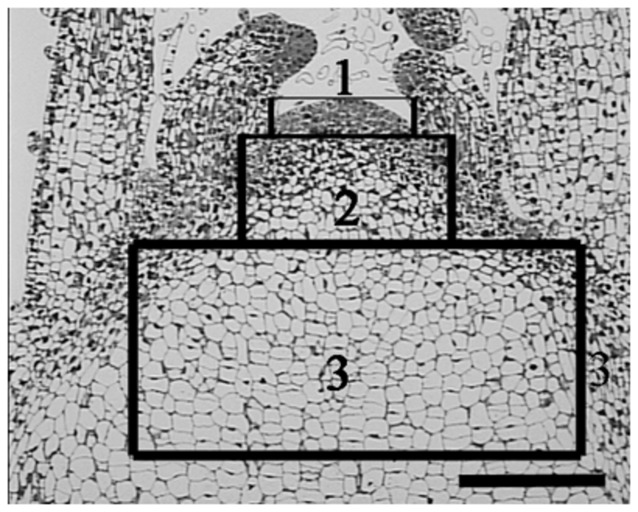
**A schematic representation of three zones of SAM used for PD ultrastructure observation and callose deposition.** Zone 1 indicates the first four layer cells in the meristem; zone 2 indicates from the fifth cell layer up to the 20^th^ cell layer (about 0.2 mm from top meristem); zone 3 indicates the cells past the 20^th^ layer. Scale bar is 200 μm.

Based on the *in situ* hybridization results of CSVd distribution in SAM of ‘Border Dark Red’ (**Figure 2C**), the SAM of both ‘Border Dark Red’ and ‘Yellow Empire’ was divided into three zones for observing ultrastructure of PD (**Figure 4**). Zone 1 indicates the first four layer cells in the meristem; zone 2 indicates from the fifth cell layer up to the 20^th^ cell layer (about 0.2 mm from top meristem); zone 3 indicates the cells past the 20^th^ layer.

**FIGURE 5:**
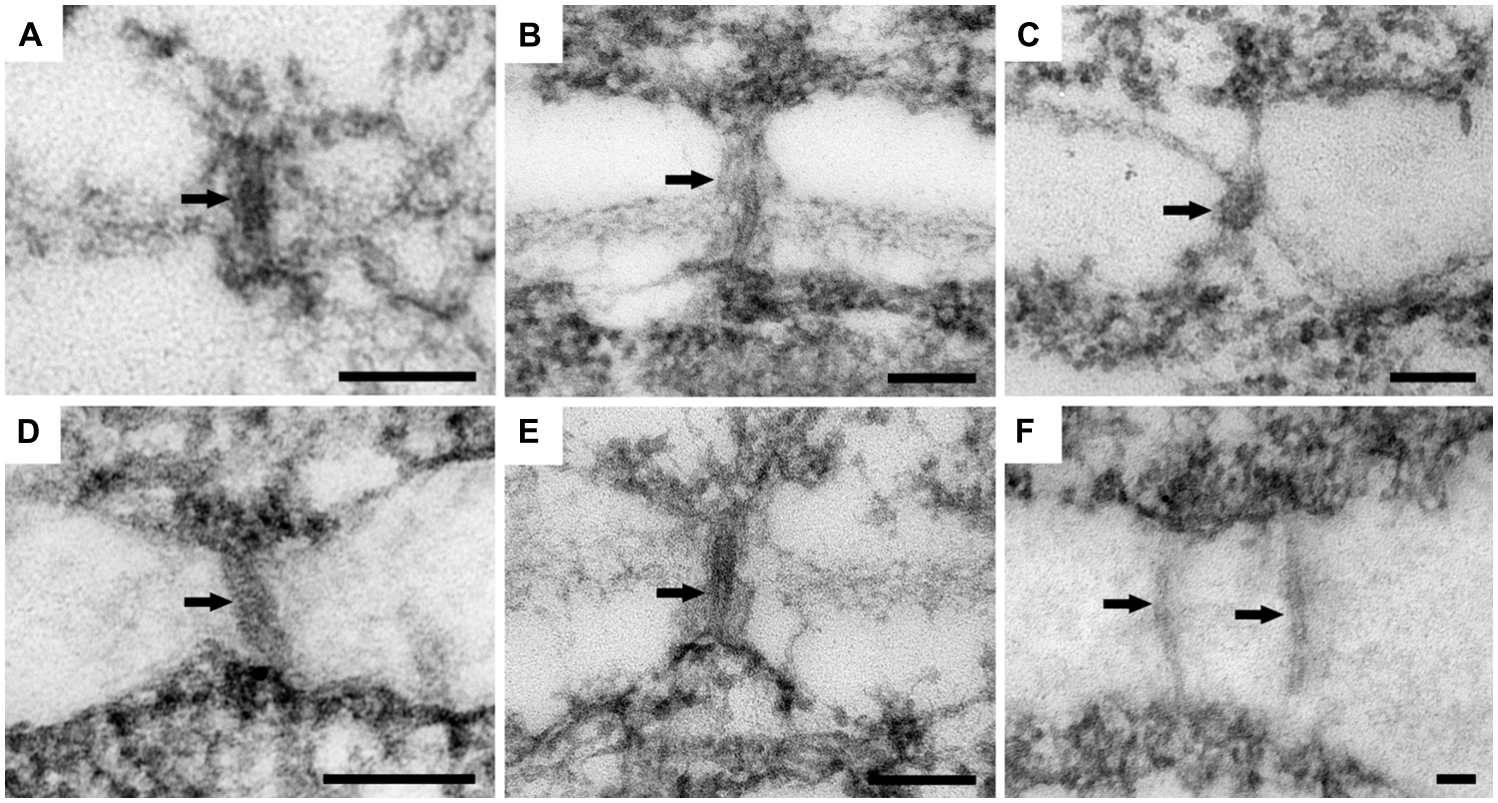
**Ultrastructure of PD in ‘Border Dark Red’ and ‘Yellow Empire’. (A–C)** are PD from zone 1, 2, and 3 of ‘Border Dark Red’. **(D–F)** are PD in zone 1, 2, 3 of ‘Yellow Empire’, respectively. Arrows indicate PD. Scale bars in **(A–F)** are 100 nm.

In ‘Border Dark Red’, although PDs were observed in all the three zones, non-branched PDs were observed to cross the cell walls in zones 1 and 2 (**Figures 5A,B**), while branched PD were observed only in zone 3 (**Figure 5C**). In ‘Yellow Empire’, the developmental pattern of PD in the three zones (**Figures 5D–F**) was quite similar to that found in ‘Border Dark Red’. In this experiment, at least 100 PDs from 50 cells of 5 SAM were observed in each of ‘Yellow Empire’ and ‘Border Dark Red’.

### CSVd INFECTION RESULTS IN AN INCREASE DEPOSITION OF CALLOSE IN THE PD OF ‘BORDER DARK RED’

**FIGURE 6:**
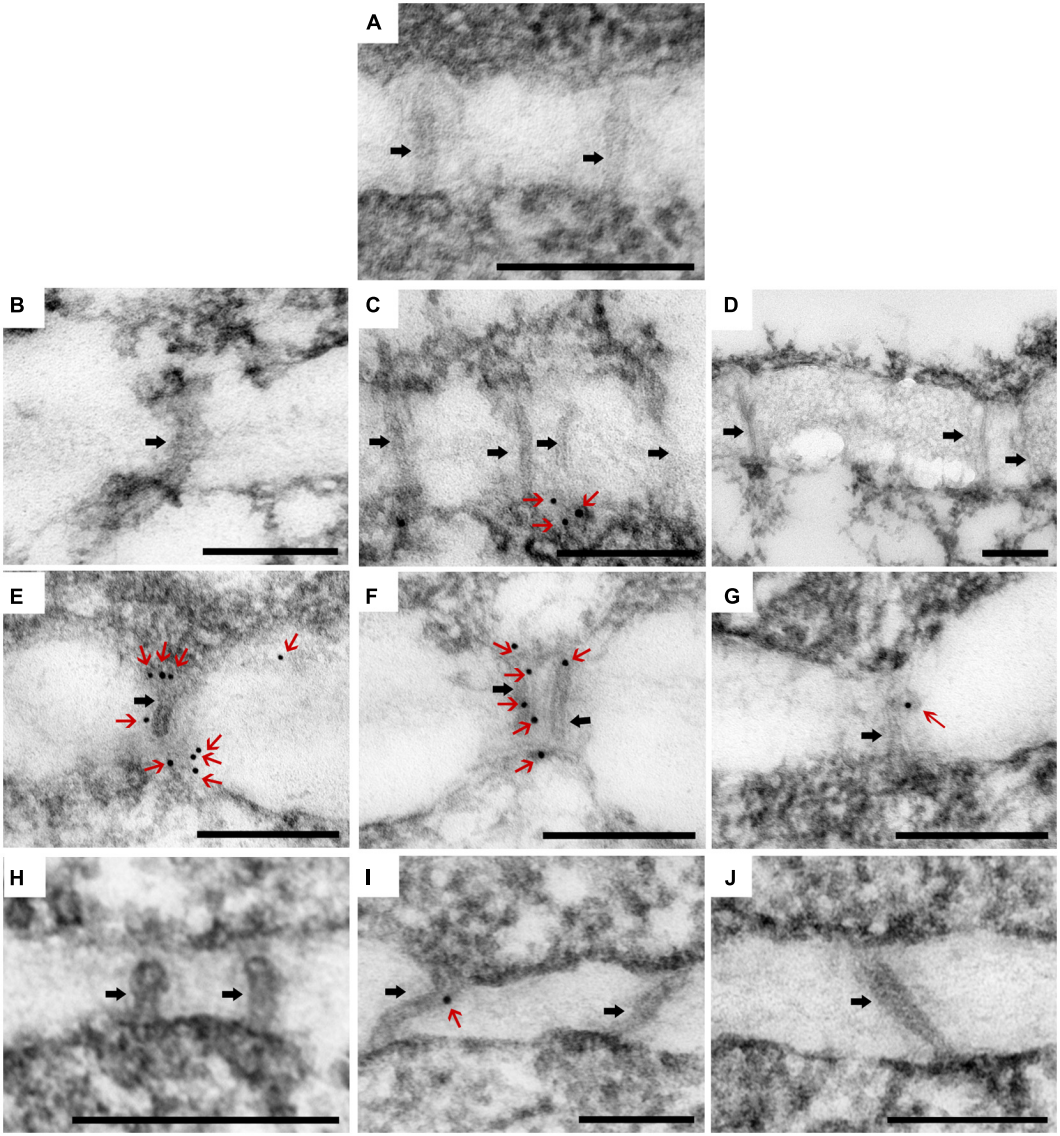
**Immunolocalization of β-1, 3-glucan (callose) in the PDs in SAM of *Argyranthemum*. (A)** Negative control.** (B–D)** Plasmodesmata (PD) of CSVd infected ‘Yellow Empire’, in zone 1, 2, 3, respectively. **(E–G)** PD of CSVd infected ‘Border Dark Red’, in zone 1, 2, 3, respectively. **(H–J)** PD of healthy ‘Border Dark Red’, in zone 1, 2, 3, respectively. Immunogold particles show β-1, 3-glucan (callose) accumulation in PD. Black arrows indicate PD. Red arrows indicate immunogold particles (callose). Scale bars of **(A–J)** are 200 nm.

Callose (β-1, 3-glucan) deposition was observed in each of the three zones of SAM (**Figure 4**), as divided for PD observation. In CSVd-infected ‘Yellow Empire’, on average less than one callose particle was found to accumulate at PD in zones 1, 2, and 3 (**Figures 6B–D**; **Table 1**). In CSVd-infected ‘Border Dark Red’, a number of callose particles (more than four) were easily seen at PD in zones 1 and 2 (**Figures 6E,F; Table 1**), while occasionally one callose particle was detected at PD in zone 3 (**Figure 6G**; **Table 1**). In the healthy ‘Border Dark Red’, callose particles were rare at PD in all the three zones (**Figures 6H–J; Table 1**). The negative control, to which no primary antibodies were added, did not show any non-specific immuno-response (**Figure 6A**). In this experiment, at least 100 PD from 50 cells of five SAM were checked in each of the three types of plants including the healthy ‘Border Dark Red’, CSVd-infected ‘Border Dark Red’ and ‘Yellow Empire’.

**Table 1:** Quantitative analysis of callose (β-1, 3-glucan) deposition at plasmodesmata (PD) in healthy ‘Border Dark Red’, and *Chrysanthemum stunt viroid* (CSVd)-infected ‘Border Dark Red’ and ‘Yellow Empire’.

	Number of gold particles per PD*		
	Healthy ‘Border Dark Red’	CSVd-infected ‘Border Dark Red’	CSVd-infected ‘Yellow Empire’
Zone 1	0.6 ± 0.1 Aa	4.0 ± 0.6 Ab	0.9 ± 0.2 Aa
Zone 2	0.5 ± 0.1 Aa	4.7 ± 0.9 Ab	0.6 ± 0.2 Aa
Zone 3	0.3 ± 0.1 Aa	1.1 ± 0.2 Ba	0.7 ± 0.2 Aa

Zone 1 contains the first four cell layers in the meristem; zone 2 the 5–20^th^ cell layer;zone 3 the cells lower than the 20^th^ cell layer in the apical dome (AD) of ‘Border Dark Red’ and ‘Yellow Empire’. Data with different upper case letters (A,B) in the same column indicate a significant difference at *p* ≤ 0.05 by the Student’s *t*-test (*n* = 30). Data with different lower case letters (a,b) in the same row indicate a significant difference at *p* ≤ 0.05 by the Student’s *t*-test (*n* = 30). *Values are means ± SE.

## DISCUSSION

CSVd can attack several plant species including *Argyranthemum* (Menzel and Maiss, 2000; Marais et al., 2011; Torchetti et al., 2012). The present study showed that CSVd infection induced obvious symptoms on greenhouse-grown ‘Yellow Empire’ and ‘Border Dark Red’ plants, including stunted growth, irregular shape of plants, flower distortion, and color breaking. Symptom development observed in the present study added valuable information for diagnosis of CSVd infection on *Argyranthemum* plants.

There have been several studies on viroid distribution in SAM of plants, for example, *Hop stunt viroid* (HSVd) in *Humulus lupulus* (Momma and Takahashi, 1983), *Potato spindle tuber viroid* (PSTVd) in *Nicotiana benthamiana* (Zhu et al., 2001; Di Serio et al., 2010), and *Peach latent mosaic viroid* (PLMVd) in *Prunus persica* (Rodio et al., 2007). In *H. lupulus*, Momma and Takahashi (1983) did not detect HSVd in 0.2 mm SAM containing AD and the first youngest two LPs in the diseased plants. In *N. benthamiana*, Zhu et al. (2001) found that PSTVd was absent in the AD, but present in tissues containing prophloem that located right below the AD. Di Serio et al. (2010) found that PSTVd could infect SAM, including upmost cell layers of AD, of RNA-dependent RNA polymerase 6-silenced *N. benthamiana* plants, but not the SAM of wild-type *N. benthamiana*. Rodio et al. (2007) reported that PLMVd was observed in the AD and in the youngest LP of *P. persica*, leaving only a few uppermost cell layers of SAM free of viroid. The distribution patterns of different viroids on the same host have also been studied. In tomato (*Solanum lycopersicum*), although both PSTVd (Qi and Ding, 2003) and *Tomato chlorotic dwarf viroid* (TCDVd; Matsushita et al., 2011) cannot invade the apical meristems, the size (in length) of viroid-free regions of SAM differed from each other: about 200 μm for PSTVd and 50 μm for TCDVd. These data indicate that the ability of viroids to invade SAM differs among viroids, and helps explain observations that meristem-based culture techniques for viroid elimination varies among viroid–host combinations (Hollings and Stone, 1970; Paludan, 1985; Paduch-Cichal and Kryczyński, 1987; Hosokawa et al., 2004b,c; Grudzińska and Solarska, 2005; El-Dougdoug et al., 2010; Savitri et al., 2013).

To date, studies on ability of the same viroid to invade SAM of different cultivars of a given plant species have never been done, and therefore, explanation as to why viroid-free frequency produced by the same method differs among plant cultivars has remained unclear. In the present study, we employed four *Argyranthemum* cultivars, and demonstrated that the ability of CSVd to invade SAM differed among *Argyranthemum* cultivars. These results may have answered the question why use of the same size of meristems resulted in different viroid-free frequencies using meristem-based methods.

Viroids move over short distance within plants by cell to cell trafficking through PD (Lucas, 1995; Ding et al., 1997; Oparka, 2004; Benitez-Alfonso et al., 2010) and for long distance through the sieve elements of the phloem (Lucas et al., 2001; Zhu et al., 2001), eventually resulting in systemic infection of the whole plant. In the present study, a similar development pattern of vascular tissues in SAM was found in both ‘Yellow Empire’ and ‘Border Dark Red’: absence of vascular tissue initiation in the uppermost layer cells of AD, presence of proxylem and prophloem in LP2, and xylem and phloem in LP3. Developmental pattern of PD was also found similar in SAM of these two cultivars. PD developed even in the uppermost layer cells, and zones 1 and 2 contained non-branched PDs, while zone 3 had branched PD. Experimental data obtained here indicate that developmental patterns of vascular tissues or PD were not likely to be responsible for causing differences in ability of CSVd to invade SAM of *Argyranthemum* cultivars.

Callose, a polysaccharide in the form of β-1, 3-glucans with some β-1, 6-branches, has been implicated in plant defenses against pathogens (Epel, 2009). To date, most studies focused on virus-infected plants (Hiruki and Tu, 1972; Beffa et al., 1996; Iglesias and Meins, 2000; Li et al., 2012), and there are few reports on viroids (Rizza et al., 2012).

An earlier study (Hiruki and Tu, 1972) found that callose accumulated at PD of non-necrotic cells adjacent to the necrotic lesions in the red kidney bean (*Phaseolus vulgaris*) that was resistant to *Potato virus M* (PVM) at 3–4 days post inoculation. Iglesias and Meins (2000) found that enhanced callose deposition delayed cell-to-cell trafficking of *Tobacco mosaic virus* (TMV) and *Potato virus X* (PVX) in a β-1, 3-glucanase-deficient mutant of tobacco. More recently, Li et al. (2012) carried out a comprehensive study investigating the effect of callose deposition at PD on cell-to-cell movement of virus, using the soybean cv. Jidou 7 (*Glycine max*), which was resistant to *Soybean mosaic virus* (SMV) strain N3, while susceptible to SMV strain SC-8. They found that the virus spread systemically throughout the plant of the soybean cv. Jidou 7 in 42 h post-inoculation by SMV strain SC-8, while SMV strain N3 was detected only at 96 h post-inoculation within a 1 mm boundary of inoculation. In the former case, callose was visible only at 2–8 h post-inoculation in the cell wall and in cytoplasm but not at PD. The authors believed that these calloses observed were likely due to mechanical damage caused by inoculation or cutting during sampling. In the latter case, callose was localized at PD, and the number of callose particles increased at 72 h post-inoculation and reached a maximum by 96 h post-inoculation. All these studies suggest that callose deposition at PD can limit or prevent the spread of viruses in resistant plants.

Working on *Citrus exocortis viroid* (CEVd), Rizza et al. (2012) found that callose depositions occurred in the phloem fibers of leaves of pre-symptomatic and symptomatic viroid infected plants, but not in those of the healthy controls. In the present study, significantly fewer callose particles were detected at PD in the three zones of SAM in the healthy ‘Border Dark Red’. In the diseased ‘Border Dark Red’, a number of callose deposits were observed at PD in zones 1 and 2, where CSVd was not able to invade. In contrast, less callose were detected in zone 3, where CSVd was abundantly present. In the diseased ‘Yellow Empire’, less than one callose particle was observed in zones 1, 2 and 3, in which all tissues were infected by CSVd. Based on these data, we assume that the number of callose particles deposited at PD is most likely responsible for differences in ability of CSVd to invade SAM among *Argyranthemum* cultivars.

It has been suggested that PD function could be regulated by callose deposition (Xu and Jackson, 2010; Faulkner and Maule, 2011). Callose deposition at PD was reported to compress the plasma membrane inward, thus creating a narrowed neck region, which in turn reduced the free space available for the passage of molecules through PD (Radford et al., 1998; Bilska and Sowiński, 2010). Callose deposition at PD was found to cause the closure of PD, while its degradation resulted in the re-opening of PD (Ruan et al., 2004). Therefore, callose deposition at PD may form a physical barrier to restrict cell-to-cell movement of viroids, as widely suggested for virus (Beffa et al., 1996; Iglesias and Meins, 2000; Li et al., 2012).

In conclusion, results obtained in the present study showed that CSVd induced obvious symptoms on greenhouse-grown *Argyranthemum* plants. The variations of distribution patterns of CSVd in SAM may resolve the question of why meristem-based methods to produce viroid-free plants have resulted in different viroid-free frequencies among different cultivars. Callose deposition at PD may restrict cell-to-cell movement of CSVd and is most likely responsible for causing differences in the ability of CSVd to invade SAM of different *Argyranthemum* cultivars.

## Conflict of Interest Statement

The authors declare that the research was conducted in the absence of any commercial or financial relationships that could be construed as a potential conflict of interest.

## ABBREVIATIONS

AD: apical dome
CSVd: Chrysanthemum stunt viroid
DIG: digoxigenin
LP: leaf primordium
PD: plasmodesmata
RT-PCR: reverse transcription polymerase chain reaction
SAM: shoot apical meristem.
